# The Role of Baseline Vagal Tone in Dealing with a Stressor during Face to Face and Computer-Based Social Interactions

**DOI:** 10.3389/fpsyg.2017.01986

**Published:** 2017-11-28

**Authors:** Daniele Rigoni, Francesca Morganti, Paride Braibanti

**Affiliations:** Department of Human and Social Sciences, University of Bergamo, Bergamo, Italy

**Keywords:** cardiac vagal tone, autonomic nervous system, respiratory sinus arrhythmia (RSA), social engagement system (SES), stress, social interaction

## Abstract

Facing a stressor involves a cardiac vagal tone response and a feedback effect produced by social interaction in visceral regulation. This study evaluated the contribution of baseline vagal tone and of social engagement system (SES) functioning on the ability to deal with a stressor. Participants (*n* = 70) were grouped into a minimized social interaction condition (procedure administered through a PC) and a social interaction condition (procedure administered by an experimenter). The State Trait Anxiety Inventory, the Social Interaction Anxiety Scale, the Emotion Regulation Questionnaire and a debriefing questionnaire were completed by the subjects. The baseline vagal tone was registered during the baseline, stressor and recovery phases. The collected results highlighted a significant effect of the baseline vagal tone on vagal suppression. No effect of minimized vs. social interaction conditions on cardiac vagal tone during stressor and recovery phases was detected. Cardiac vagal tone and the results of the questionnaires appear to be not correlated. The study highlighted the main role of baseline vagal tone on visceral regulation. Some remarks on SES to be deepen in further research were raised.

## Introduction

Cardiac vagal tone is the output of the central autonomic network and, according to the neurovisceral integration model (Thayer et al., 2009), it may be considered a marker of the ability to face stress. The relationship between cardiac vagal tone it’s role in understanding the intersubjective communication that involves social engagement behaviors has also been studied, in particular by the polyvagal theory (Porges, 1995, 2003, 2007; Appelhans and Luecken, 2006; Chambers and Allen, 2007). In particular, research on stress conducted on new-borns and infants constituted some of the first studies in the field of cardiac vagal tone during the 1970 and 1980s (Porges, 2007). In line with this tradition of research, Polyvagal theory proposes a new conceptualization of stress and considers cardiac vagal tone as an effective index of stress and stress vulnerability (Porges, 1995).

Stress reactions to challenge and stress vulnerability can be effectively measured through the respiratory sinus arrhythmia (RSA), that is a measure of heart rate variability (HRV) tied to the respiratory cycle. RSA is determined by vagal pathways originating in the nucleus ambiguous, and it can thus be considered a measure of cardiac vagal tone (Porges, 1995). The dynamic regulation of the “vagal brake” during various tasks and challenges can be accurately assessed by measuring RSA (Porges, 1995, 2007).

Research on regulatory control and coping with stress in college student samples has generally highlighted a negative correlation between high cardiac vagal tone and the levels of negative emotional arousal in response to daily life stressors for moderate to high-intensity stressors (Fabes and Eisenberg, 1997). Furthermore, stressors based on cognitive tasks that require focused attention have been found to cause a modulation in HRV. Research has highlighted that people with a greater baseline HRV had faster reaction times and higher levels of variation by reducing cardiac vagal tone during attention tasks (Porges, 1972, 1973, 2007). Positive relations between low defensiveness and higher cardiac vagal tone and between higher behavioral activation and higher cardiac vagal tone were also found (Movius and Allen, 2005).

Polyvagal theory, unlike other theories on the role of cardiac vagal tone in emotion and self-regulation (e.g., the neurovisceral integration model proposed by Thayer et al., 2009), focuses also on social aspects linked to self-regulation processes. Through hypothesizing a link between visceral homeostasis and social behavior, the theory posits that a particular branch of the vagus nerve—the myelinated vagus, originating in the nucleus ambiguous—can have an inhibiting effect on the sinoatrial node functionality contributing in that way to the processes involved in emotion regulation and facilitating social engagement. It assumes that humans can inhibit more primitive neural structures that control fight, flight or freezing behaviors through more recently developed neural structures, such as the myelinated vagus (Porges, 2001, 2007).

At last, the theory also introduces the construct of social engagement ssystem (SES), which is defined by polyvagal theory as a complex system of anatomical and physiological linkages between the vagal regulation of the heart through the myelinated ventral vagus and the striated muscles of the face, head and neck (Porges, 2001, 2007). Several studies on cardiac vagal tone and SES focused on the degree to which people experience positive emotions and social connectedness. Main relevant findings of this kind of studies highlighted that people with higher baseline vagal tone experienced higher levels of positive emotions and displayed a pattern of reciprocal effects between positive emotions, social engagement and cardiac vagal tone (Kok and Fredrickson, 2010). The parasympathetic influence on the heart was positively associated with engagement coping and aspects of social well-being (Geisler et al., 2013). People exposed to interventions that positively affect vagal regulation (like, for example, a loving kindness meditation training) also experienced higher levels of positive emotions and more supportive social interactions (Kok et al., 2013). Other studies have focused directly on the relation between cardiac vagal tone and social engagement behaviors in an experimental setting. For example, in a study conducted by Sgoifo et al. (2003) a correlation between a low baseline vagal tone and submissive patterns of interaction was found in two different types of interviews.

A recent meta-analysis considered data from 14 different studies that measured HRV on adolescents (participants aged more than 12 years) and adults that were participating in different experimental procedures involving social interactions. This meta-analysis outlined that HRV patterns of change during social interaction are mediated by the valence of the administered social task. Negative social interactions decrease HRV in a manner comparable to the Trier Social Stress Task (considered to be a well-established social stressor task, generally used in experimental procedures) although dyadic interactions of a positive or neutral valence were not found to significantly change HRV from baseline. Different results were found in clinical populations included in the meta-analysis (Shahrestani et al., 2015).

There is instead a paucity of studies that have focused on the analysis of the direct relationship between cardiac vagal tone and the functioning of other components of the SES (Kettunen et al., 2000; Demaree et al., 2006). In this area of research the relationship between baseline vagal tone and facial valence related to negative stimuli (Demaree et al., 2006) was analyzed. Moreover, the relationship between the baseline vagal tone and the activation of facial expressions during the Rorschach test (Kettunen et al., 2000) was observed recording interbeat intervals, electrodermal activity, and facial electromyography during the experiment. The pattern of facial responding was not found to be the result of an active response modulation process (Demaree et al., 2006), coherently with polyvagal theory, that considers vagal regulation to be influenced by a process of neuroception that is not under voluntary control (Porges, 2007). Although, on a limited sample of 37 adult male participants the regulation of the vagal system was found to be linked to facial muscle activity. The baseline and task-induced levels of RSA were also positively related to expressive responses with a positive change in mood and more variability in the emotional experience during tasks (Kettunen et al., 2000). In particular this latter study seems to confirm the link between vagal regulation and SES functioning proposed by the polyvagal theory.

Polyvagal theory also proposes that stimulation and activation of the somatomotor components of the SES (e.g., the muscles of face and head) could trigger a regulation of the visceral components of the SES (Porges, 2007) and highlights a bidirectional link between physiological regulation and social engagement behaviors. From this point of view social behaviors could influence autonomic regulation and sustain or impede social engagement strategies, depending on triggered visceral states.

Together, previous studies suggest a link between cardiac vagal tone and social engagement behaviors. Unfortunately previous research hasn’t generally studied the cardiac vagal modulation elicited by the same stressful task administered with different social interaction conditions in an experimental setting. The present study aims at considering also this aspect and the possible feedback effect produced by SES functioning on visceral regulation.

The present study aims to consider the effect of vagal regulation during a stressful situation, focusing distinctly on the role of baseline vagal tone and on the role of the feedback effect produced by social interaction though the SES functioning. In particular the vagal regulation during a cognitive stressor provided in two different modalities was observed. Accordingly, a minimized social interaction group (MinSI) underwent the experimental procedure while interacting with a personal computer, and a social interaction group (SI) underwent the procedure while interacting with the experimenter. The experimental procedure was designed for both groups to determine a stressful effect on participants like that induced by a similar experimental task used in previous studies (e.g., the task used in the research conducted by Movius and Allen, 2005). The valence of the task used in this study in both groups can be classified as negative, based on the point of view expressed by Shahrestani et al. (2015) because it elicits a stress reaction in participants, although this effect could be lighter than other negative tasks (e.g., like those based on disagreements). The SI group’s procedure was designed to assess the possible effect on participants’ vagal regulation produced by a human social interaction in administering the same stressful task as that proposed to MinSI group. Possible differences in vagal modulation registered on SI group compared to MinSI group are supposed to be linked to the feedback effect produced by social interaction between the experimenter and participants produced by elements present in SI group but not in MinSI group (like face to face interaction effect, human prosody compared to an automatic computer processed voice, physical proximity and hand and arm’s movements).

### Main Hypothesis

It was hypothesized that participants with a higher RSA baseline would also be characterized by a higher RSA suppression (RSA baseline minus RSA during stressful task) while facing the stressful task and by a higher RSA recovery (RSA after a relaxation procedure minus RSA during a stressful task) after the stressful task. This hypothesis is coherent with Polyvagal theory (Porges, 1995, 2003) that considers higher baseline vagal tone as an index of a higher capacity to adequately modulate cardiac vagal tone during stress and to recover more efficiently from stress (e.g., Katz and Gottman, 1997).

It was also hypothesized that participants in SI group, who underwent the stressor and relaxation exercises while interacting with the experimenter, will have lower vagal suppression during the stressor and lower vagal recovery after the stressor compared to participants in MinSI, who underwent the entire procedure while interacting with a personal computer. This hypothesis is based on the possible effect of social behaviors as potential regulators of autonomic activity and visceral states through the somatomotor components of the SES (Porges, 2007).

Moreover, possible correlations were hypothesized between baseline vagal regulation and anxiety, anxiety in social interactions and emotion regulation styles. Specifically, a negative correlation was hypothesized between RSA baseline and trait-state anxiety, and a negative correlation between RSA baseline and social interaction anxiety. It is plausible that the correlation between poor vagal regulation and higher levels of anxiety found in people with anxiety disorders (e.g., Lyonfields et al., 1995) can also be extended to non-clinical sample as in previous research (e.g., Movius and Allen, 2005). A positive correlation was also hypothesized between RSA baseline and emotion regulation style based on reappraisal. This hypothesis is based on the positive correlation between better vagal regulation, the ability to experience positive emotions and effective social engagement (Porges, 2003; Kok and Fredrickson, 2010). Moreover, participants with a low RSA were hypothesized to be less relaxed during the experience than subjects with a high RSA baseline. At last, participants in SI group are expected to report a feeling of judgment or feel tenser than participants in MinSI group. Other differences in the results of the three semantic continuums proposed in a debriefing questionnaire (from “tense” to “relaxed,” from “bored” to “interested,” from “feeling judged” to “not feeling judged”) will also be expected.

## Materials and Methods

### Participants

Participants were undergraduates students enrolled in a psychology course at the University of Bergamo. They were recruited randomly from a list of volunteers who want to receive university credits for participating the research. Participants who had a history of cardiac disease or who were currently taking cardiovascular medications were ruled ineligible for the study. Moreover, participants were asked to avoid coffee, cigarettes, and alcohol assumption before experiment. A total of 70 subjects (mean age ± SD, 22.01 ± 3.48) contacted by e-mail agreed to participate in the study. All participants were females because there were very few male students available on the list to guarantee a balanced mixed sample for the experiment.

### Instruments

In this study, the recording of HRV data was done with a Polar RS800CX (Polar Electro Oy, Kenpele, Finland) heart rate monitoring system at 1000 Hz, as in other previous published studies (e.g., Quintana et al., 2012a). This instrument wirelessly receives data from a chest strap (two-lead) worn by participants. Notwithstanding the fact that there has been some discussion about the validity of Polar to measure R-R intervals (Quintana et al., 2012b; Wallen et al., 2012), recent research has highlighted that the Polar heart rate monitor and ECG can be used interchangeably in healthy people because of the high intra-class correlation coefficient between the recording done simultaneously with ECG and with the Polar heart rate monitor on the same healthy people. Moreover, the Bland–Altman limits of the agreement method demonstrated an excellent level of agreement between the measures collected with the two different instruments (Weippert et al., 2010).

The self-report questionnaires included the Italian versions of State and Trait Anxiety Scales (STAI Y Forms 1 and 2; Spielberger et al., 1983; Pedrabissi and Santinello, 1996), the Social Interaction Anxiety Scale (SIAS; Mattick and Clarke, 1998; Sica et al., 2007) and the Emotional Regulation Questionnaire (ERQ; Gross and John, 2003; Balzarotti et al., 2010). The STAI Y Form 1 and STAI Y Form 2 questionnaires respectively evaluated state anxiety and trait anxiety. The SIAS questionnaire assessed how people handle distress during social interactions. The ERQ, that consists of two scales, measured emotions expressive suppression and cognitive reappraisal. The STAI Form 2 for trait anxiety was completed at the beginning of the experiment, before the RSA baseline recording. The remaining questionnaires were completed after the last physiological recording.

### Procedures

After signing a consent form, participants were asked to complete the questionnaire and they subsequently prepared for psychophysiological recording. Irrespectively of the answers given on the questionnaires, the participants were randomly assigned to two different groups:

• Minimized Social Interaction (MinSI): participants underwent the procedure while interacting with a personal computer. The computer communicated the instructions during both the experimental task and the guided relaxation procedure through an audio message previously recorded using a computerized humanoid-like voice.
• Social Interaction (SI): participants underwent the procedure administered by an experimenter who directly read the same instructions used for participants in the MinSI group during both the experimental task and the following relaxation procedure. The experimenter looks to the participants during the experimental task and tries to keep as much as possible a neutral, non-supportive. At the same time the experimenter takes a non-judgmental attitude toward participants, and keeps a marked and not chaotic rhythm in administering the arithmetic task maintaining a posture not pushed out toward the participants or too recoiled from them.

The physiological recording phase starts after the participants’ assignment to the experimental group. After being instructed on how to correctly wear the chest strap, participants remained seated for a few minutes in a room that contained a table and two chairs. Participants were then asked to remain seated for 5 min to record “some physiological parameters at rest.” Interbeat intervals (IBIs) recorded during this 5 min period yielded the RSA baseline (as in Movius and Allen, 2005). Participants were breathing spontaneously during this period without controlling for respiration because according to Denver et al. (2007) the controlling for breathing means also removing a relevant factor that influence HRV, at least for resting states.

After the RSA baseline was collected, participants were subjected to the stressor task. In both groups, participants were asked to perform serial paced arithmetic counting backward in varying intervals, starting with a four-digit number for 10 30-s periods to induce attention-focusing activity (Sloan and Bigger, 1991). This stressor task was introduced by replicating and slightly varying with different time periods serial-paced arithmetic similar to that used in a previous study by Movius and Allen (2005). Participants in the MinSI group performed serial-paced arithmetic while reading the instructions written on paper and interacting only with an artificial humanoid-like voice, created using open-source software (eSpeak – www.espeak.sourceforge.net). Participants in the SI group performed serial-paced arithmetic while interacting with the experimenter, who read the instructions with participants and guided them by word of mouth through the serial-paced arithmetic with the same timing and arithmetic structure as in the MinSI group. The experimenter looks to the participants while they were performing their arithmetic task and he kept as much as possible a neutral facial expression not answering participants’ requests for support. For both groups, to increase the stressful effect of the task, the serial-paced arithmetic instructions stated that the participants’ performance would be subsequently checked for accuracy after the experiment, before assigning them the expected university credits (two credits for fewer than five mistakes, one credits for more than five mistakes).

Finally, participants in both groups participated in a guided relaxation exercise adapted from the mindfulness body scan tradition (Zindel et al., 2013). Following the relaxation exercise, IBIs were recorded for 5 min.

Then participants completed a paper and pencil questionnaire packet and were involved in a debriefing session. For both groups, during the debriefing, participants were asked to express a subjective evaluation on an explorative self – report questionnaire composed by a seven-point Likert scale of the stressor task difficulty (“Was the arithmetic task easy or difficult?”), of their personal efficacy during the stressor task (“Did you feel effective in facing the arithmetic task?”), of the relaxation procedure (“Did you find the relaxation procedure relaxing or not relaxing?”) and of their personal ability to relax during the relaxation procedure (“Did you feel relaxed during the relaxation procedure?”). At last, participants were asked to express their personal evaluation of the entire experimental experience on three continuums: from tense to relaxed, from bored to interested and from feeling judged to feeling not judged. Questionnaires to evaluate body mass index (BMI) and regular intensive sport activities were also completed by the experimental groups.

At the end of the experiment, the main objective of the study was explained to participants and they were thanked for their participation.

### Data Reduction and Analysis

Raw data from Polar RS800CX were exported as text files through the Polar Protrainer software. They were then imported into ARTiiFACT software version 5.0, a tool for heart rate processing and heart rate variability analysis (Kaufmann et al., 2011). This open-source software allows for correcting IBIs raw data for potential artifacts using an artifact-detection algorithm developed in 1990 by Bertson, Quigley, Jang, and Boysen based on individual threshold criteria of artificial beats (Kaufmann et al., 2011). The software also allows for a complementary visual manual inspection of the raw data. Detected artifacts were corrected using cubic spline interpolation. Corrected IBIs were then exported from ARTiiFACT as a text file and imported into CMetX software (Allen et al., 2007). CMetX software allows for obtaining an estimate of RSA converting the IBI series to a time series sampled at 10 Hz, filtering the series using a 241-point optimal finite impulse response digital filter and then taking the natural log of the variance of the filtered waveform as the estimate of RSA. CMetX allows for the extraction of heart period variability in the high-frequency band (0.12–0.4 Hz) and for producing an estimate of RSA that correlates 0.99 with that produced by MX Edit Software^1^ (Allen et al., 2007).

## Results

Descriptive statistics of the main collected measures are presented in **Table 1**. Four RSA parameters were collected: RSA baseline (at rest), RSA stressor (during the task), RSA relaxation (during relaxation), and RSA post-relaxation (at the end of the relaxation). Moreover, three measures were considered indexes of vagal activity and regulation: RSA at baseline, RSA suppression (derived from RSA baseline minus RSA during the stressor task) and RSA recovery (derived from RSA after relaxation minus RSA during the stressor task). Higher RSA suppression expresses a temporary deactivation of the vagal brake on the sinoatrial node during the stressor, and higher RSA recovery indicates a more intense reactivation of the vagal control of the heart after the stressor.

**Table 1:** Means (±SD) for total sample in MinSI and SI groups.

	Total sample	MinSI	SI
RSA baseline	6.1 (1.1)	6.0 (1.2)	6.2 (0.9)
RSA stressor	5.3 (1.4)	5.0 (1.4)	5.6 (1.4)
RSA relax	6.4 (1.6)	6.3 (1.9)	6.5 (1.1)
RSA post-relax	6.2 (0.9)	6.2 (0.8)	6.2 (0.9)
STAI Y Form 2	45.0 (8.7)	43.8 (8.8)	46.1 (8.8)
STAI Y Form 1	42.6 (12.8)	41.9 (13.5)	43.2 (12.2)
SIAS	26.9 (14.7)	26.3 (14.7)	27.6 (15.0)
ERQ suppression	12.6 (5.9)	13.1 (5.7)	12.1 (6.2)
ERQ reappraisal	27.9 (7.2)	28.3 (6.3)	27.4 (8.0)

*RSA baseline (RSA at rest); RSA stressor (RSA during stressor); RSA relax (RSA during relaxation); RSA post-relax (RSA after relaxation); STAI Y Form 2 (Trait Anxiety); STAI Y Form 1 (State Anxiety); SIAS (Social Interaction Anxiety Scale); ERQ Suppression (Emotion Regulation Questionnaire – Suppression Scale); ERQ Reappraisal (Emotion Regulation Questionnaire – Reappraisal Scale)*.

### Baseline Vagal Tone and Vagal Regulation during the Experimental Procedure

Participants’ baseline vagal tone was classified as high vagal tone (HVT) if their RSA at baseline was higher than the median of the whole sample or low vagal tone (LVT) if their RSA at baseline was lower than the median of the sample. The BMI and the level of sport activity of the subjects were checked. RSA baseline was not found to be correlated to BMI or sport activity levels.

A repeated-measures ANOVA performed for RSA levels during the four phases of the experiment (baseline, stressor, relaxation, post-relaxation) highlighted a RSA modulation according to the four experimental phases [*F*(3,64) = 13.11, *P* < 0.01].

Respiratory sinus arrhythmia modulation during the four phases was affected by the interaction between phases and baseline vagal tone [*F*(3,64) = 4.74, *P* < 0.01] and was not affected by the interaction between phases and the condition (assignment to MinSI/SI groups) or by the interaction between the phases and baseline vagal tone and condition.

**FIGURE 1:**
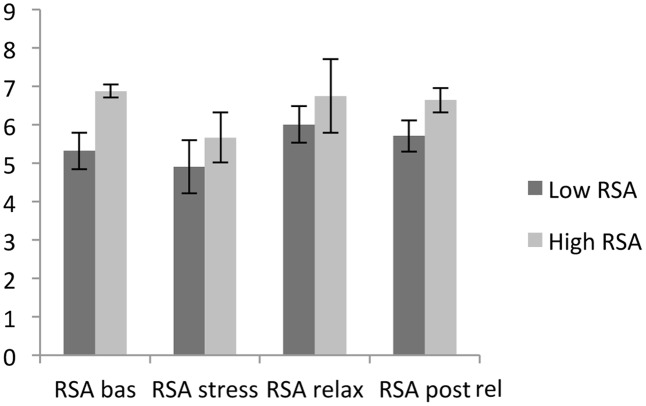
Respiratory sinus arrhythmia (RSA) average levels during the four phases of the experiment. RSA bas (RSA baseline); RSA stress (RSA stressor); RSA relax (RSA relaxation); RSA post rel (RSA post-relaxation).

For HVT and LVT participants, there were significant differences in RSA baseline [*t*(43) = -9.16, *P* < 0.05] and the RSA stressor [*t*(68) = -2.38, *P* < 0.05]. The same result was found for RSA relaxation [*t*(50) = -2.03, *P* < 0.05] and RSA after relaxation [*t*(64) = -5.30, *P* < 0.05]. The average RSA levels during the four phases of the experiment are represented below in **Figure 1**.

During the four phases, the RSA baseline was significantly differentiated from the RSA stressor [*t*(69) = 5.18, *P* < 0.05], the RSA stressor was discriminated from RSA relaxation [*t*(69) = -5.34, *P* < 0.05], and the RSA stressor was differentiated from RSA post-relaxation [*t*(69) = -5.74, *P* < 0.05]. For both HVT and LVT participants, the RSA baseline average levels were higher than the average RSA stressor level, the average RSA stressor level was lower than the RSA relaxation level, and the average RSA stressor level was lower than the average RSA post-relaxation level.

**FIGURE 2:**
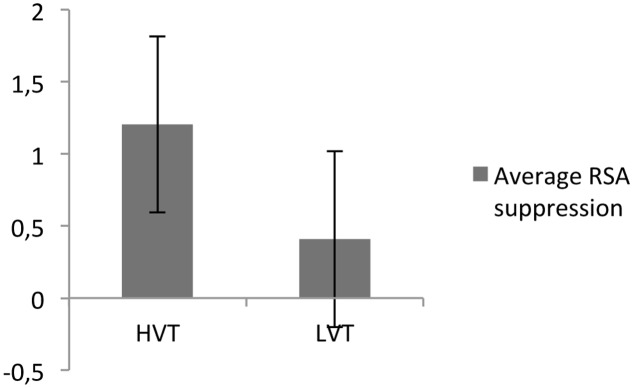
Respiratory sinus arrhythmia suppression for participants with high (HVT) and low (LVT) RSA baselines.

Finally, the RSA baseline had a significant effect on RSA suppression [*t*(68) = -2.67, *P* < 0.05; see **Figure 2**].

### Baseline Vagal Tone and Social Interaction

Given that RSA modulation was not affected by the interaction between phases and the condition (assignment to MinSI/SI groups) or by the interaction between the phases, the baseline vagal tone and the condition, it was not possible to find significant differences in vagal regulation for participants in SI group compared to MinSI group as hypothesized.

For further information we can anyway report that *t*-tests highlighted that the RSA stressor was different for the MinSI and SI conditions [*t*(68) = -2.06, *P* < 0.05] Also RSA recovery was different for participants in the MinSI and SI conditions [*t*(68) = 2.01, *P* < 0.05]. These latter differences are anyway not significant at statistical level considering that RSA modulation was not affected by the interaction between the phases, baseline vagal tone and the assignment to MinSI/SI groups as highlighted before.

### Baseline Vagal Tone, Anxiety, and Emotion Regulation Style

No significant correlation between RSA baseline and state or trait anxiety, as measured by STAI Y Forms 1 and 2, was found in this experiment. RSA baseline was not also found to relate to scores on social interaction anxiety, as measured by the SIAS (Social Interaction Anxiety Scale). Moreover, no significant correlation was found between RSA baseline and the score registered for the Suppression and the Reappraisal scales in ERQ.

### Baseline Vagal Tone, Group Assignment, and Participants’ Subjective Perceptions

No significant difference in answering the debriefing items was found based on the baseline vagal tone or on the assignment to experimental conditions. The participants’ perception of the task difficulty, their personal efficacy during arithmetic task, the efficacy of the relaxation procedure and their personal ability to relax were not different between the HVT and LVT participants; nor between participants assigned to the MinSI or SI conditions. Participants in both groups perceived the arithmetic task as more “difficult” than “not difficult,” they perceived themselves more as “not effective” than “effective” in accomplishing the arithmetic task, the relaxation procedure was considered more “relaxing” than “not relaxing” and participants felt themselves as “more relaxed” than “not relaxed.” The same result was found regarding the self-positioning on the three experiential dimensions (from “tense” to “relaxed,” from “bored” to “interested” and from “feeling judged” to “not feeling judged”). Participants felt tenser than relaxed, slightly more interested than bored and slightly more “not judged” than “judged.”

## Discussion

The main aim of the present paper was to study the role of baseline vagal tone and of the feedback effect of SES functioning on the cardiac vagal tone modulation and on the ability to face with stress. The results of the present study highlighted a modulation of the vagal tone linked to the stressor and an effect of the baseline vagal tone on the suppression of vagal activity during the stressor. The effect of the baseline vagal tone on the ability to regulate themselves while facing a stressor is clearly highlighted by these results and consistent with polyvagal theory and an extensive literature on the field (e.g., Fabes and Eisenberg, 1997; Movius and Allen, 2005; Appelhans and Luecken, 2006).

The hypothesized effects of the baseline vagal tone level on RSA recovery were instead not found in this study. RSA changes were not affected by the interaction between phases and by the assignment to MinSI/SI groups. The interaction between the phases, baseline vagal tone and group conditions, contrary to our hypothesis didn’t reveal significant differences in vagal regulation for participants in SI group compared to MinSI. These results can be explained firstly considering that RSA regulation could be mediated mainly by the particular valence of the task as highlighted by the meta-analysis by Shahrestani et al. (2015). A possible feedback effect on participants’ SES, if actually present, could be not always present for all participants or too light to make a difference in SI group compared to MinSI group. This point give rise to possible questions about the link between vagal regulation and SES functioning and about the specific characteristics that a social interaction has to present in order to influence vagal regulation and SES functioning. More experimental research on these points seems to be therefore desirable.

Another possible explanation could be linked to the role of neuroception in social interactions: it can greatly vary on a subjective level. The different characteristics in a social interaction pattern can be assessed as safe by a subject and dangerous by other subjects (or as safe in a particular situation and not safe in another one by the same subjects). A different neuroception in our participants could have led to different vagal regulation reactions and this phenomenon may have affected participants’ individual vagal regulation by producing no difference in vagal regulation in SI group compared to MinSI group. Accordingly, a pioneering study testing if psychometric variables (such as anxiety traits and resiliency disposition) can predict the recovery from decreased hearth period after stress, showed that cardiac vagal tone and resilience interacted synergistically in the promotion of stress recovery highlighting the often underestimated role played by individual differences in determining the effect of arousal and emotional modulation (Souza et al., 2007).

We have also to consider that some researchers in particular in the field of cognitive science and artificial intelligence (Dennet, 1987) have hypothesized that people can confer also to computers and other technological devices a kind of intentional stance comparable to that present in human interactions. Hypothetically also this aspect could be evaluated to explain the absence of significant differences between autonomic regulation in SI group and in MinSI group.

In summary the role of baseline vagal tone on the ability to regulate cardiac vagal tone while facing a stressor is clearly highlighted by the results of the present study. The complex link between vagal tone modulation, SES functioning and social behaviors seems to be instead an area that needs more in-depth analysis.

Concerning the emotional correlates of cardiac vagal tone, no significant correlations were found between RSA measures and anxiety/social interaction/emotion regulation questionnaires. These results are in a large part coherent with previous literature where the expected correlations between cardiac vagal tone and anxiety or emotion regulation were not found (Movius and Allen, 2005). However, in this study, participants with low levels of social anxiety showed better modulation of cardiac vagal tone when facing a stressor and subjects with higher motivation and lower defensiveness revealed higher vagal tone. It is therefore necessary to evaluate whether correlations between RSA and anxiety and between RSA and emotion regulation styles are present only in clinical samples or if those results could be linked to some specific individual characteristics (e.g., to non-clinical personality traits). Further investigation with wider samples could better investigate this point.

At last, the results registered through the debriefing section highlighted that overall participants reported they were tenser than relaxed, slightly more interested than bored and more not judged than judged during the experiment. This result is coherent with the effect on vagal regulation during the experiment. The fact that participants felt more not judged than judged is in line with our purpose to keep neutral the attitude of the experimenter toward participants. These subjective evaluations between participants did not vary between the MinSI and SI conditions. However, it must also be considered that autonomic regulation patterns measured through RSA could be related also to psychological aspects that are not easily accessible to conscious awareness, particularly when defensive fight/flight or freezing strategies are activated. These patterns are also less accessible to a conscious assessment when they become a usual reaction pattern for a person in interactive situations. This subject should be further investigated to better understand the links between sensorimotor, emotional and cognitive levels of experience.

## Conclusion

First of all it is necessary to focus on the complexity of the concept of “social interaction” that we tried to assess in this study. Given this premise, based on the theoretical point of view proposed by polyvagal theory (Porges, 2007) we considered the “social interaction” factor by distinguishing two different elements that can characterize it: (a) the feedback effect that a human social interaction between the experimenter and participants can determine on the visceral states and autonomic regulation of participants through SES and (b) the possible effect on autonomic regulation linked to the innate tendency to co-regulate with others as outlined by an extensive literature on intersubjectivity (Morganti et al., 2008). The absence of significant differences in autonomic regulation between MinSI and SI groups registered in this study stress the need for an in-depth definition of the role of social factors and SES functioning in autonomic regulation.

First, the role of the experimenter and his no-judging behavior, requested in the present study, probably relies on the experimenter’s personal ability to keep this attitude. A video recording of social interaction in SI group would provide us with more elements useful to evaluate non-verbal patterns exhibited by experimenter during the interaction with participants. Furthermore, a rule that request participants to keep their gaze toward the experimenter during the experimental tasks should be introduced in the procedure. Some participants, in fact, maintained their gaze toward the table during the arithmetic task, avoiding the experimenter’s gaze and thus ignoring his feedback. Moreover, we did not consider in this study measurements concerning social support, social connectedness and the ability to feel positive emotions. It would be important to introduce these measurements in future experimental research and also to assess possible different effects on vagal regulation linked to a momentary exposure to a certain type of social interaction compared to the continuous exposure to a consolidated pattern of social interaction.

Another possible limitations of this study is the number of participants in the two groups and the possible difficulties in effectively assessing and evaluating RSA differences in a between subject designed experiment (Quintana and Heathers, 2014) can be addressed. About this latter point, we followed recommendations provided by these authors (Quintana and Heathers, 2014) for between subjects design, adopting a sample size between 30 and 77 subjects and groups with more than the minimum of 20 participants.

An important element to take into account is also linked to the kind of experimental sample we considered: it is in fact necessary to study in more detail possible differences assessable in clinical samples compared to not clinical ones like the one considered in the present study. When studying the SES impairments in particular, polyvagal theory generally considers clinical populations (e.g., people with reactive attachment disorder, autism, or post-traumatic stress disorder), where a low RSA baseline and poor vagal regulation can generally be found (Porges, 2003, 2007). Based on the results highlighted by Shahrestani et al. (2015) in studies on clinical samples they considered in their meta-analysis, with clinical populations in fact it would be possible to find a different kind of vagal regulation while facing a stressor in a social condition like that proposed in SI group. For these people or in general for people with a very low RSA level we can in fact hypothesize a lack of vagal flexibility in facing a stressor which could probably make a significant difference in vagal regulation between SI group and MinSI group. This study was instead conducted with a non-clinical sample. In the present sample, the average RSA baseline was 6.1 (*SD*: 1.0), which is not comparable with the RSA level registered in clinical populations such as a sample of people with post-traumatic stress disorder with an RSA baseline average level of 4.87 (*SD*: 1.26; Sahar et al., 2001). It is therefore possible that a stronger effect of a neutral social engagement realized in the SI condition could be observed in a clinical sample.

Given the results of the present study some possible areas of further investigation, could be to reach a definition of social engagement not only in terms of the functioning of its constituent components (or in terms of emotional and social effects distinctly) but also with a focus an enactive and embodied perspective linked to vagal regulation patterns. This perspective could take into account the synchrony and rhythms in interaction, which are at the basis of the embodied interaction (Morganti et al., 2008). Future research will focus on the relationship between embodied interactive behaviors and vagal regulation. Social engagement behaviors – in terms of dyadic interactions and their negotiation, of non-verbal communication, of posture, of facial expressions and of gesture quality – should then be adequately considered. Thus, these types of parameters could be used to design experimental models of interaction that can correctly discriminate the different patterns of social engagement.

It could be also important to evaluate the role of SES functioning and social engagement behaviors in facing a stressor that has a more evident and stronger social dimension. It would be important for example to understand whether these results would be registered for the SI condition using a different type of stressor, such as socially stressing interviews or experimental procedures based on role playing, where patterns of social and embodied interactions could be more evident than with a cognitive stressor.

In conclusion the present research highlights an important role of baseline vagal tone in the ability to face a stressor through an efficient RSA suppression. Coherent with the recent research in the field, our study appears to reaffirm the importance of considering RSA as a marker of stress and stress vulnerability within a social engagement. In our opinion, polyvagal theory and research on autonomic regulation will introduce new research questions and will facilitate a deeper understanding of the role of this hierarchical and evolutionary interpretative model for physiological regulation in social engagement or defensive behaviors. It will also shed new light on the understanding of social behaviors in different settings, providing a method to understand social interactions by integrating sensorimotor, emotional and cognitive points of view. Probably a productive dialog between polyvagal theory and embodied theories can bring more light to the coregulation processes in social interaction and could further contribute to an in-depth analysis of the previously highlighted areas of research. A more integrated way that brings together sensorimotor, regulatory, emotional and cognitive aspects, that are generally addressed from separate points of view, will be useful in many different psychological realms where social interaction is the core element to analyze. It also will provide a theoretical background for different applications aimed at developing better regulation, social interaction and communication between people.

## Ethics Statement

This study was carried out in accordance with the recommendations of University of Bergamo with written informed consent from all subjects. All subjects gave written informed consent in accordance with the Declaration of Helsinki. The protocol was approved by the University of Bergamo research office.

## Author Contributions

All the authors contributed to the conception and design of study, as to the analysis and interpretation of data. DR conducted the experiment and acquired data. DR and FM drafted the manuscript, all the authors revised the manuscript critically for important intellectual content and approved the submitted version.

## Conflict of Interest Statement

The authors declare that the research was conducted in the absence of any commercial or financial relationships that could be construed as a potential conflict of interest.

## Footnotes

1 Delta – Biometrics, Inc., 1988–1993.
